# MicroRNA-145 Impairs Classical Non-Homologous End-Joining in Response to Ionizing Radiation-Induced DNA Double-Strand Breaks via Targeting DNA-PKcs

**DOI:** 10.3390/cells11091509

**Published:** 2022-04-30

**Authors:** Muddenahalli Srinivasa Sudhanva, Gurusamy Hariharasudhan, Semo Jun, Gwanwoo Seo, Radhakrishnan Kamalakannan, Hyun Hee Kim, Jung-Hee Lee

**Affiliations:** 1 Laboratory of Genomic Instability and Cancer Therapeutics, Cancer Mutation Research Center, Chosun University School of Medicine, 375 Seosuk-dong, Gwangju 61452, Korea; sudhan.molbio@gmail.com (M.S.S.); haribiochem08@gmail.com (G.H.); semojun@chosun.ac.kr (S.J.); revival_k@naver.com (G.S.); kmknphd@gmail.com (R.K.); kimhhee13@gmail.com (H.H.K.); 2 Department of Cellular and Molecular Medicine, Chosun University School of Medicine, 375 Seosuk-dong, Gwangju 61452, Korea

**Keywords:** classical non-homologous end-joining pathway (c-NHEJ), DSBs, DNA-PK_cs_, microRNA

## Abstract

DNA double-strand breaks (DSBs) are one of the most lethal types of DNA damage due to the fact that unrepaired or mis-repaired DSBs lead to genomic instability or chromosomal aberrations, thereby causing cell death or tumorigenesis. The classical non-homologous end-joining pathway (c-NHEJ) is the major repair mechanism for rejoining DSBs, and the catalytic subunit of DNA-dependent protein kinase (DNA-PK_cs_) is a critical factor in this pathway; however, regulation of DNA-PK_cs_ expression remains unknown. In this study, we demonstrate that miR-145 directly suppresses DNA-PK_cs_ by binding to the 3′-UTR and inhibiting translation, thereby causing an accumulation of DNA damage, impairing c-NHEJ, and rendering cells hypersensitive to ionizing radiation (IR). Of note, miR-145-mediated suppression of DNA damage repair and enhanced IR sensitivity were both reversed by either inhibiting miR-145 or overexpressing DNA-PK_cs_. In addition, we show that the levels of Akt1 phosphorylation in cancer cells are correlated with miR-145 suppression and DNA-PK_cs_ upregulation. Furthermore, the overexpression of miR-145 in Akt1-suppressed cells inhibited c-NHEJ by downregulating DNA-PK_cs_. These results reveal a novel miRNA-mediated regulation of DNA repair and identify miR-145 as an important regulator of c-NHEJ.

## 1. Introduction

Faithful inheritance of genetic information from one generation to the next and adaptation to a steadily changing environment are two sides of the same coin, reflecting both genetic stability and genetic modification that must be maintained in parallel to ensure the survival of both parent and offspring. Thus, DNA-modifying agents can be either a threat leading to extinction or a driving force for evolution, depending on how DNA lesions are addressed [1]. DNA double-strand breaks (DSBs) are regarded as one of the most deleterious outcomes of exposure to DNA-damaging agents, whether endogenous or environmental [2]; eukaryotes also generate this type of DNA lesion as part of their normal physiological processes, such as those that occur during B-cell or T-cell development [1,2,3,4]. To counteract these DSBs, cells have evolved several repair methods, including classical non-homologous end-joining (c-NHEJ), homologous recombination (HR), alternative-end joining (a-EJ), and single-strand annealing (SSA) [1]. While the high fidelity of HR guarantees genomic integrity, the other three methods are prone to errors to varying degrees and tend to cause chromosomal alterations. Therefore, properly coordinated regulation of these repair pathways, in accordance with the particular type of DSB, is critical for maintaining genomic integrity and allowing modifications that are required for gene rearrangements, such as those that occur during the development of T and B lymphocytes [1]. 

c-NHEJ and HR are of particular importance in DSB repair, while c-NHEJ mainly operates throughout the cell cycle and plays a key role in the generation of functional T or B cell receptors [1,2,3,4]. HR is restricted during the S/G2 phase when a sister chromatid is available as a template for repair [5]. During c-NHEJ, DSBs are recognized and bound initially by heterodimeric KU proteins (KU70 and KU80), after which the catalytic subunit of DNA-dependent protein kinase (DNA-PK_cs_) is recruited to the broken ends of the DNA [1,6]. A deficiency in DNA-PK_cs_ causes radiosensitive severe combined immunodeficiency in humans [7], leading to similar phenotypes in mice [8] and rendering cells sensitive to ionizing radiation (IR) [9]. Interestingly, the loss of this protein has been shown to increase the frequency of HR after exposure to IR [10,11], suggesting that DNA-PK_cs_ plays an important role in the choice between the NHEJ and HR pathways in response to DSBs. However, whether cellular levels of DNA-PK_cs_ are relevant for regulation remains to be determined. 

MicroRNAs (miRNAs) are a class of short (20–24 nucleotides), noncoding RNAs that bind to the 3′-untranslated regions (3′-UTRs) of target mRNAs, leading to the suppression of protein synthesis of the corresponding genes. A growing body of evidence implicates miRNAs in the orchestration of DNA damage responses through the negative regulation of DNA repair-related proteins [12,13,14,15,16,17]. 

In this study, we established that DNA-PK_cs_ is a target for a particular miRNA, miR-145, and that downregulation of DNA-PK_cs_ by miR-145 impairs the normal c-NHEJ response to IR-induced DSBs, leading to an increased sensitivity of affected cells to IR. This defective c-NHEJ activity was rescued by co-transfection with either an antagomir of miR-145 or DNA-PK_cs_ cDNA lacking the 3′-UTR. In addition, we showed that activated Akt1 upregulates DNA-PK_cs_ expression by downregulating miR-145. Taken together, these results suggest that miR-145 plays an important role in the regulation of DNA-PK_cs_ and may therefore be a therapeutic target for radioresistance in for cancer and other diseases. 

## 2. Materials and Methods

### 2.1. Cell Culture and Treatment

The human cervix adenocarcinoma HeLa cells, the human bone osteocarcinoma U2OS cells, the human embryonic kidney HEK293T cells, the human breast cancer BT549, MCF7, MDA-MB 231, MDA-MB 453 cells, and the human prostate cancer DU145 cells were obtained from the American Type Culture Collection (ATCC, Rockville, MD, USA). Cells were cultured in Dulbecco’s modified Eagle’s medium (DMEM) (Gibco-BRL, Grand Island, NY, USA), supplemented with 10% heat-inactivated fetal bovine serum (FBS; Cambrex Corp., East Rutherford, NJ, USA), 100 units/mL penicillin, and 100 µg/mL streptomycin sulfate (Invitrogen, Carlsbad, CA, USA). All cells were maintained in a humidified incubator containing 5% CO_2_ at 37 °C. To induce DNA double strand breaks, exponentially growing cells were irradiated from ^137^Cs source (Gamma cell 3000 Elan irradiator, Best Theratronics, Ottawa, ON, Canada) at different doses depending on the types of experiments and allowed to recover at 37 °C. To inhibit Akt1 function, HeLa cells were treated with 50 μM perifosine (Sigma, St. Louis, MO, USA) for 24 h.

### 2.2. Cell Transfection

Hsa-miR-145 duplex and negative control miRNA were purchased from Bioneer (Daejeon, South Korea). Cells were transiently transfected with 50 nM miRNA using lipofectamine RNAiMax (Invitrogen, Carlsbad, CA, USA) according to the manufacturer’s recommendations. For rescue experiments, a miR-145 inhibitor (anti-miR-145) and pcDNA-DNA-PK_cs_ vector were used. For expression of Akt1, pUSE-Myc-Akt1 vector was used. pcDNA empty vector and scrambled oligonucleotide were used as negative controls.

### 2.3. Antibodies

The following antibodies were used for immunoblotting: mouse monoclonal anti-DNA-PK_cs_ (1:1000 Neo markers/Thermo scientific laboratories, Middlesex, MA, USA), monoclonal anti-DNA-PK_cs_ (1:1000; Santa Cruz Biotechnology, Santa Cruz, CA, USA), polyclonal anti-KU80 (1:1000; Santa Cruz Biotechnology), polyclonal anti-KU70 (1:1000; Santa Cruz Biotechnology), anti-GAPDH mouse monoclonal antibody (1:10,000; Santa Cruz Biotechnology), and monoclonal anti-α-tubulin (1:10,000; Santa Cruz Biotechnology). Formation of γ-H2AX foci was detected by immunofluorescence staining using a γ-H2AX mouse monoclonal antibody (JBW301, Upstate Biotechnology, Temecula, CA, USA) at a 1:200 dilution. 

### 2.4. Western-Blot Analysis

Cells were lysed in ice-cold RIPA lysis buffer: 50 mM Tris (pH 8.0) containing 150 mM sodium chloride, 1.0% NP-40 (or Triton X-100), 0.5% sodium deoxycholate, 0.1% SDS (sodium dodecyl sulfate), 2 mM EDTA, and protease inhibitor cocktail (Roche, Basel, Switzerland). Equal amounts of proteins were then resolved on 6–15% SDS-PAGE gels, followed by electrotransfer to polyvinylidene difluoride membranes (Millipore, Bedford, MA, USA). The membranes were blocked for 1 h in TBST [10 mM Tris–HCl (pH 7.4), 150 mM NaCl, 0.1% Tween 20] containing 5% skim milk at room temperature and then incubated with the indicated primary antibodies overnight at 4 °C. Membranes were washed and incubated with appropriate secondary antibodies for 2 h at room temperature and membranes were developed using enhanced chemi-luminescence detection system. The amounts of DNA-PKcs protein were quantified using Scion Image software (Scion Corp., Chicaga, IL, USA).

### 2.5. Reverse Transcription-Quantitative Real-Time PCR (RT-qPCR)

Total RNA was extracted using TRIzol reagent (Invitrogen). For quantitation of DNA-PK_cs_ mRNA, cDNA was synthesized using 1 μg total RNA, Random hexamer (Promega, Madison, WI, USA) and M-MLV reverse transcriptase (Invitrogen). Real-time PCR analysis was performed using the SYBR green-based fluorescent method (SYBR premix Ex Taq kit, Takara, San Jose, CA, USA) and the MX3000P^®^ qRT-PCR system (Stratagene, La Jolla, CA, USA) with specific primers. Primers used for real-time PCR are as follows: *DNA-PK_cs_* forward, 5′-GCCAAATCTCAGGATACAAGGA-3′ and *DNA-PK_cs_* reverse, 5′-TGGTTCTCAGCTTAGTTATTGGTG-3′. To quantify miR-145, cDNA was synthesized using Mir-X^TM^ miRNA first-strand synthesis and SYBR qRT-PCR kit (Takara) according to the manufacturer’s instructions. Has-miR-145-MI0000461 was used as primer for real-time qPCR. The quantity of transcripts was calculated based on the threshold cycle (C_t_) using the delta-delta C_t_ method that measures the relative of a target RNA between two samples by comparing them to a normalization control RNA (*gapdh* or U6). 

### 2.6. MicroRNA Luciferase Reporter Assay

Segments of the 3′UTR of DNA-PKcs containing putative miR-145 binding sites were cloned into pMIR-REPORT *firefly* luciferase vector (Applied Biosystems, Waltham, MA, USA). Deletion mutants of predicted miR-145 binding sites were made using the GENEART Site-Directed Mutagenesis kit (Invitrogen). For the luciferase activity assay, pMIR-REPORT luciferase vectors containing wild type or mutant 3′UTRs of DNA-PKcs and pRL-TK vector containing *Renilla* luciferase as a transfection control were co-transfected into HeLa cells using Lipofectamine 2000 (Invitrogen), and subsequently, the same cells were transfected with miR-145 and/or anti-miR-145. After 24 h of transfection, the luciferase assay was performed using the dual luciferase reporter assay system (Promega) according to the manufacturer’s instructions. Luciferase activity was quantified using a luminometer (Glomax, Promega).

### 2.7. Immunofluorescence Cell Staining

To visualize γ-ray-induced γ-H2AX foci, cells cultured on coverslips were washed twice with PBS and fixed in 100% ice cold methanol for 10 min, followed by permeabilization with 0.3% Triton X-100 for 15 min at room temperature. Next, the coverslips were washed three times with PBS, followed by blocking with 0.1% bovine serum albumin in PBS for 1 h at room temperature. The cells were immunostained using primary antibody directed against the γ-H2AX proteins overnight at 4 °C. After 3 times being washed with PBS, the cells were stained with the appropriate secondary antibodies conjugated with Alexa Fluor 488- or Alexa Fluor 594 (green and red fluorescence, respectively; Molecular Probes, Eugene, OR, USA). The coverslips were mounted onto the slides using Vectashield mounting medium containing 4′, 6-diamidino-2-phenylindole (DAPI; Vector Laboratories, Burlingame, CA, USA). Fluorescence images were taken under a confocal microscope (Zeiss LSM 510 Meta; Carl Zeiss, Jena, Germany) and analyzed with Zeiss LSM Image Examiner software (Carl Zeiss). Percentage was calculated among at least 100 cells by dividing the number of γ-H2AX foci-positive cells by the number of DAPI-stained cells.

### 2.8. NHEJ Assay

The NHEJ assay was measured in HeLa and U2OS cells stably expressing EJ5-GFP, using methods previous described [18,19]. To generate stable EJ5-HeLa and EJ5-U2OS cells, cells were transfected with pimEJ5-GFP plasmids (#44026 addgene, Watertown, MA, USA) using Lipofectamine 2000. 24 h after transfection, 5 μg/mL of puromycin was added to the media and cultured for 2 weeks for selection. Stable cells were confirmed by expression of GFP after I-SceI transfection. EJ5-HeLa and EJ5-U2OS cells were transfected with miR-145 or a control miRNA using lipofectamine RNAiMAX, and then transfected with pCBA-I-SceI vector. After 2 days, the percentage of GFP-positive cells which had repaired the DSBs generated by I-SceI was determined by flow cytometry (FACSCalibur, BD Bioscience, San Jose, CA, USA). The acquired data was analyzed using CellQuest Pro software (BD Biosciences). 

### 2.9. HR Assay

HR was measured using DR-GFP-U2OS cells as described previously [19,20]. Briefly, DR-GFP U2OS cells were transfected with control miRNA or miR-145 using lipofectamine RNAiMAX (Invitrogen). At 24 h after transfection, pCBA-I-SceI plasmids were transfected into DR-GFP U2OS cells. GFP-positive cells which had repaired by HR were counted 48 h after transfection using flow cytometry (FACSCalibur). The data was analyzed by CellQuest Pro Software (BD Bioscience). 10,000 cells were analyzed and repeated three times.

### 2.10. In Vitro NHEJ Assay 

The pEGFP-Pem1-Ad2 plasmid was digested with HindIII restriction endonuclease to remove Ad2 and generate staggered ends. A supercoiled pEGFP-Pem1 vector was used as a positive control for standardization of transfection and analysis conditions. The pCMV-dsRed-express plasmid (Takara) was co-transfected into HeLa or U2OS cells with either linearized pEGFP-Pem1-Ad2 or supercoiled pEGFP-Pem1 as a control for transfection efficiency [21]. In a typical reaction, 5 × 10^2^ cells were transfected with supercoiled or linearized pEGFP-Pem1-Ad2, together with pDsRed2-N1 plasmid, using Lipofectamine 2000 (Invitrogen) according to the manufacturer’s recommended protocol. Forty-eight hours after transfection, green (EGFP) and red (DsRed) fluorescence were measured by flow cytometry (FACSCalibur, BD Biosciences).

### 2.11. Single-Cell Gel-Electrophoresis (Comet Assay)

Double strand brake (DSB) repair was visualized by neutral single-cell agarose-gel electrophoresis, as described previously [22]. Briefly, HeLa or U2OS cells transiently transfected with miR-145, anti-miR-145, a control miRNA, or the DNA-PK_cs_ cDNA were irradiated with γ-ray of 5 Gy dose, followed by incubation in culture medium at 37 °C for appropriate lengths of time. Cells were then harvested (~10^5^ cells per pellet), mixed with low melting agarose, and layered onto agarose-coated glass slides. The slides were maintained in the dark for all of the remaining steps. Slides were submerged in lysis solution (10 mM Tris-HCl (pH 10), 2.5 M NaCl, 0.1 M EDTA, 1% Triton X-100, 10% dimethyl sulfoxide) for 1 h and incubated for 30 min in neutral electrophoresis solution (100 mM Tris, 300 mM Sodium Acetate at pH 9.0). After incubation, slides were electrophoresed (~30 min at 1 V/cm tank length), air-dried, neutralized, and stained with SYBR green. Average comet tail moment was scored for 40–50 cells/slide using a computerized image analysis system (Komet 5.5; Andor Technology, South Windsor, CT, USA).

### 2.12. Clonal Survival Assay

After treatment with γ-ray irradiation at different dose, 5 × 10^2^ cells were immediately seeded onto a 60-mm dish in duplicate and grown for 2–3 weeks at 37 °C to allow colony formation. Colonies were stained with 2% methylene blue in 50% ethanol and counted. The fraction of surviving cells was calculated as the ratio of the plating efficiencies of treated cells to untreated cells. Cell survival results are reported as the mean value ± SD for three independent experiments. 

### 2.13. Statistical Analyses

Unpaired Student’s *t*-test was used to test for Statistical comparisons of data. The Data are presented as the mean ± SD. A *p* value < 0.01 was considered to be statistically significant. GraphPad Prism (GraphPad Software, San Diego, CA, USA) and Excel (Microsoft, Redmond, WA, USA) were used for the analyses.

## 3. Results

### 3.1. miR-145 as a DNA-PK_cs_-Regulating miRNA 

**Figure 1:**
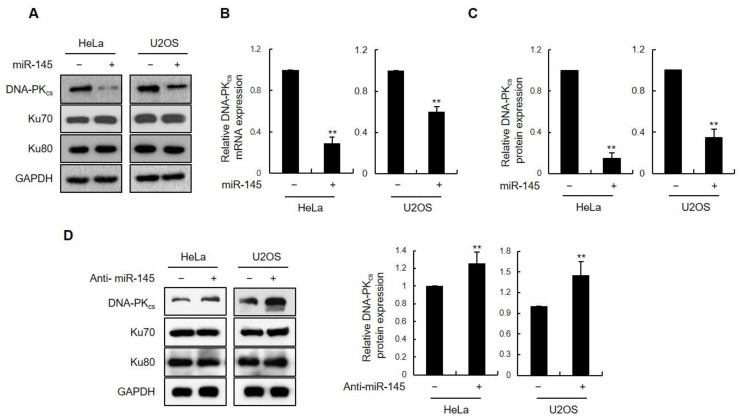
miR-145 negatively regulates DNA-PK_cs_ expression in human cells. (**A**) Western blot analysis of DNA-PK_cs_ expression in HeLa and U2OS cells 48 h after transfection with miR-Ctrl or miR-145. Ku70 and Ku80 protein levels were measured as negative controls. (**B**) Relative expression levels of DNA-PK_cs_ mRNAs were detected 48 h post-transfection and quantified using quantitative RT-PCR. The RT-PCR signal is normalized to that of the housekeeping gene GADPH. Data represent the mean ± standard deviation (SD) (*n* = 3); **, *p* < 0.01. (**C**) Quantification of DNA-PK_cs_ western blot signals from three independent experiments as performed in (**B**) using Sicon image software. DNA-PK_cs_ protein levels were normalized using Ku70 protein levels (control). Data represent the mean ± SD (*n* = 3); **, *p* < 0.01. (**D**) Western blot analysis of DNA-PK_cs_ levels in U2OS and HeLa cells 48 h after transfection with the control or miR-145 inhibitor. The band intensities of DNA-PK_cs_ and Ku70 immunoblots were quantified using Sicon image software and the relative DNA-PK_cs_ protein levels were normalized to those of the control and represented as mean values from three independent experiments.

To determine whether miRNAs are involved in regulating DNA-PK_cs_ expression, we scanned the six most commonly used miRNA databases (TargetScan 7.0, miRWalk, miRanda, miRDB, miRMap, and RNAhybrid) to identify those with the potential to target the 3′-UTR of DNA-PK_cs_ mRNA. Thirty miRNAs were consistently identified across all the six databases (Supplementary Table S1). Among these, only three (has-miR-145-5p, has-miR-150-5p, and has-miR-488-5p) had a total context score of –0.25 or lower, as calculated by TargetScan 7.0, and were further considered (Supplementary Table S2) [23]. Each of the three candidate miRNAs were screened for gene silencing effects on DNA-PK_cs_ expression following transient transfection into HEK293T cells. Western blot analysis showed that miR-145 had the strongest suppressive effect on DNA-PK_cs_ expression but had no effect on the expression of other NHEJ factors, such as Ku70 and Ku80 (Figure S1A). Quantitative RT-PCR analysis revealed decreased levels of DNA-PK_cs_ transcripts, indicating that high miR-145 levels cause DNA-PK_cs_ mRNA destabilization (Figure S1B). miR-145 overexpression also reduced DNA-PK_cs_ protein and mRNA expression in HeLa and U2OS cells (Figure 1A,B). Quantification of DNA-PK_cs_ immunoblots from three independent experiments using both HeLa and U2OS cells confirmed a robust reduction in DNA-PK_cs_ protein levels (Figure 1C). Conversely, we observed a slight increase in DNA-PK_cs_ protein levels upon transfection of HeLa and U2OS cells with the miR-145 inhibitor (Figure 1D).

### 3.2. The DNA-PK_cs_ 3′UTR Is Directly Targeted by miR-145

**Figure 2:**
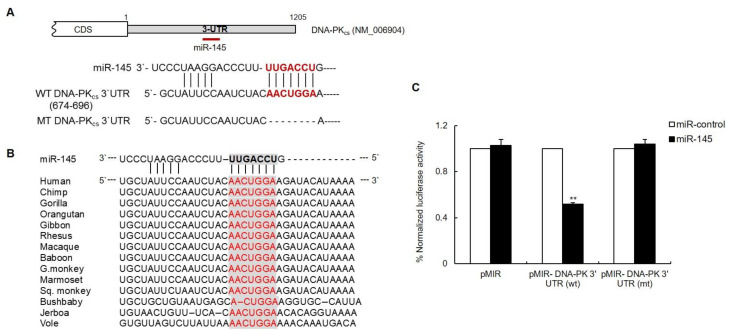
The 3′-UTR of DNA-PK_cs_ is targeted by miR-145. (**A**) A schematic diagram of the predicted miR-145-binding sites in the 3′-UTR of DNA-PK_cs_ mRNA. MT-DNA-PK_cs_ has a deletion of seven bases at the 3′-UTR (red). (**B**) Alignment of DNA-PK_cs_ 3′UTR sequences from fourteen vertebrate species. A conserved miRNA-mRNA interaction site, identified based on a seed sequence complementary to miR-145 nucleotides 1–8 (8-mer), is highlighted using gray boxes. (**C**) Luciferase reporter vectors carrying wild type or mutant 3′UTR of DNA-PK_cs_ were co-transfected with either miR-145 or a control miRNA into HeLa cells. Luciferase activity was measured 24 h after transfection. Data is represented as the mean ± SD (*n* = 3); **, *p* < 0.01.

Sequence analysis of the DNA-PK_cs_ 3′-UTR using the target prediction methods of the above-mentioned six algorithms revealed one putative miR-145 binding site located between nucleotides 674 and 696 on the 3′-UTR of human DNA-PK_cs_ mRNA (Figure 2A and Supplementary Figure S2). Moreover, multiple sequence alignment of the DNA-PK_cs_ 3′-UTR from different vertebrate species indicated conservation of the miR-145 target region, especially at the miR-145 seed-matched sites (Figure 2B). To demonstrate that miR-145 directly targets the 3′UTR of DNA-PK_cs_, this region, including the predicted binding site, was cloned downstream of a luciferase reporter gene and co-transfected into HeLa cells along with either miR-145 or a negative miR-control containing a scrambled sequence. As shown in Figure 2C, a significant decrease in luciferase activity was observed in miR-145-transfected cells as compared to that in the control cells. Notably, no significant change in luciferase activity was observed in the presence of a vector in which the putative seed region of miR-145 was deleted. These results support the conclusion that miR-145 negatively modulates DNA-PK_cs_ expression by directly binding to its 3′-UTR.

### 3.3. miR-145 Causes an Accumulation of Unrepaired DSBs after IR and Affects Cell Survival

**Figure 3:**
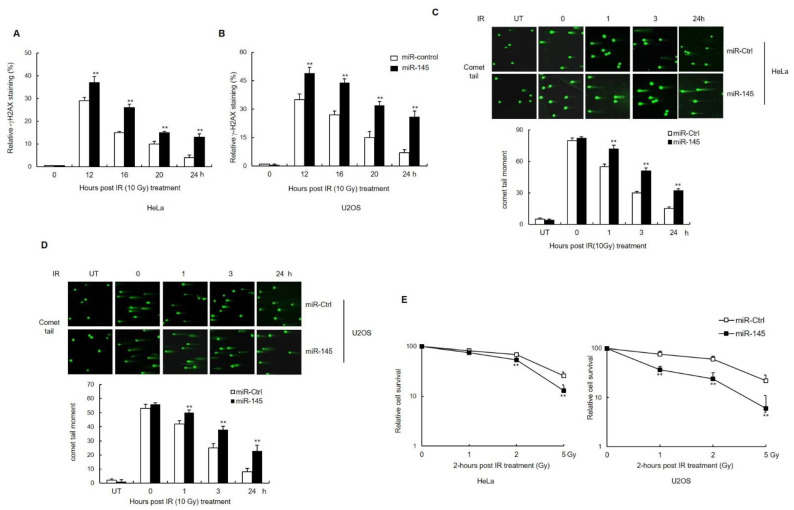
Effect of miR-145 overexpression on DNA repair and cell radiosensitivity. (**A**,**B**) HeLa (**A**) and U2OS (**B**) cells transfected with either miR-145 or miR-Ctrl were irradiated with 10 Gy and then fixed for immunofluorescence staining of γ-H2AX at the indicated time points. The percentage of γ-H2AX foci-stained cells was calculated. (**C**,**D**) HeLa (**C**) and U2OS (**D**) cells transfected with either miR-145 or miR-Ctrl were irradiated with 10 Gy and then subjected to a neutral comet assay at the indicated time points. Representative blots are shown. The comet tail moment was counted, and approximately 100 cells were analyzed in each group. (**E**,**F**) HeLa (**E**) and U2OS (**F**) cells transfected with miR-145 or a control miRNA were irradiated with γ-ray at indicated doses, seeded onto dishes, and grown for 2–3 weeks. Viability of treated cells was examined using the clonogenic survival assay. In all panels, mean ± SD is shown. **, *p* < 0.01 versus control group. IR: irradiation.

Suppression of NHEJ-mediated DNA repair results in higher levels of unrepaired, damaged DNA, which can be assayed by measuring the levels of γ-H2AX that persist after DNA damage [24,25]. HeLa or U2OS cells expressing miR-145, along with the respective control cells, were exposed to IR for 12, 16, 20, and 24 h and stained for assessing γ-H2AX levels. Twelve hours after irradiation, both HeLa and U2OS cells overexpressing miR-145 had significantly more foci than the respective control cells. After 24 h, most of the-H2AX foci were resolved in control cells, but a significant amount of γ-H2AX remained in the cells overexpressing miR-145 (Figure 3A,B and Supplementary Figure S3). To examine DSB repair more directly, the same set of cells was treated with IR and subjected to the comet assay. At 3 h after IR treatment, DNA comet tail moments were significantly extended in cells transfected with miR-145 compared to those in the control cells (Figure 3C,D). We then tested the effect of miR-145 overexpression on cell survival after IR exposure. Again, either HeLa or U2OS cells were transfected with either the control or miR-145, and clonogenic survival was measured following IR treatment. We found a significant reduction in survival following irradiation when miR-145 was overexpressed (Figure 3E,F). Similar to clonogenic survival, the MTT assay showed that the cell viability of miR145-overexpressing cells was reduced after treatment with neocarzinostatin (NCS), which mimics DSB (Supplementary Figure S3C). Collectively, these findings suggest that miR-145 inhibits DSB repair, which likely contributes to the observed IR sensitization phenotypes. 

### 3.4. miR-145 Reduces the DSB Repair by Downregulating DNA-PK_cs_

**Figure 4:**
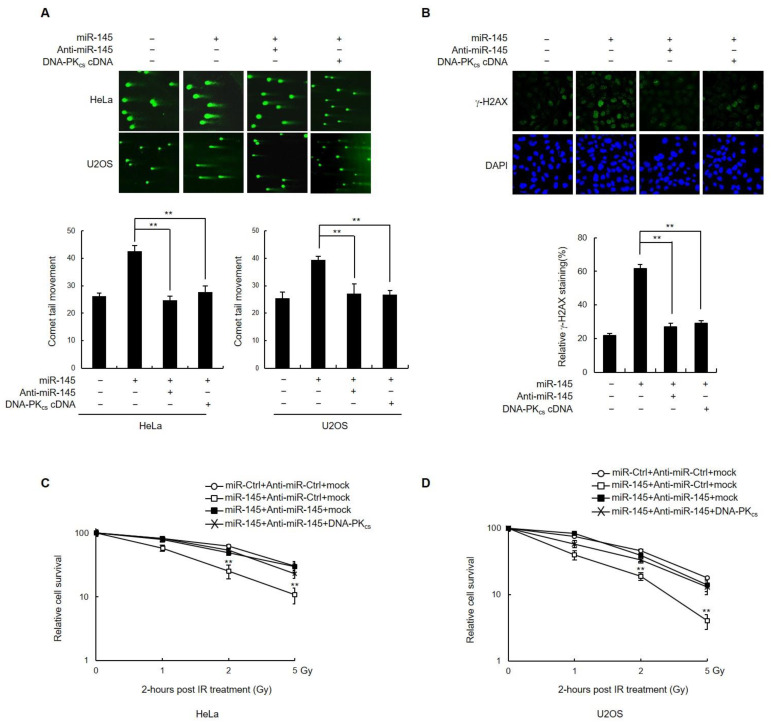
Expression of anti-miR-145 or miR-145-insensitive DNA-PK_cs_ cDNA rescues DNA repair defect and radiosensitivity. (**A**) HeLa and U2OS cells were co-transfected with combinations of miR-145, miR-Ctrl, anti-miR-145, or DNA-PK_cs_ cDNA as indicated. Three hours after γ-ray irradiation with 10 Gy, the cells were subjected to the neutral comet assay. Representative images (top) and quantification (bottom) of comet tail moment are shown. (**B**) HeLa cells co-transfected with either miR-145 or miR-Ctrl, as well as either anti-miR-145 or DNA-PK_cs_ cDNA, were γ-ray irradiated with 10 Gy and subjected to immunofluorescence staining for γ-H2AX at the 20 h time point. (**C**,**D**) HeLa (**C**) and U2OS (**D**) cells co-transfected with combinations of miR-145, miR-Ctrl, anti-miR-145, or DNA-PK_cs_ cDNA were γ-ray irradiated at the indicated doses, cultured for 2–3 weeks, and subjected to the clonogenic survival assay. In all panels, mean ± SD is shown. **, *p* < 0.01 versus control group.

The next question was whether inhibiting miR-145 would return DNA-PK_cs_ to normal expression levels and rescue DSB repair. As expected, transfection of anti-miR-145 in either HeLa or U2OS cells expressing miR-145 restored DNA-PK_cs_ protein levels to those of the control (Figure S4A). To determine whether inhibition of miR-145 in these cells also affected DSB repair, the persistence of DNA damage was measured using the comet assay 3 h after IR treatment. The results indicated that co-expression of anti-miR145 effectively reversed the effects of miR145 overexpression on the comet tail moment (Figure 4A). Similarly, the percentage of cells with γ-H2AX foci at 20 h after IR was much lower in cells expressing the miR-145 inhibitor, indicating that DSB repair was restored to normal (Figure 4B). 

To further validate the idea that miR-145 disrupts DSB repair through the downregulation of DNA-PK_cs_, we tested whether overexpression of DNA-PK_cs_ lacking a 3′-UTR could rescue the DSB repair deficiency that results from the overexpression of miR-145. Both HeLa and U2OS cells were co-transfected with DNA-PK_cs_ along with either control or miR-145 (Figure S4B), after which they were treated with IR and subjected to both comet assay and γ-H2AX staining to measure the levels of DSB damage. The results indicated that overexpression of DNA-PK_cs_ lacking a 3′-UTR restored both the comet tail moment (Figure 4A, last lane) and γ-H2AX foci formation (Figure 4B, last lane) to control levels. Similarly, the negative effect of miR-145 on the survival of HeLa and U2OS cells after exposure to γ-ray irradiation was reversed when either anti-miR-145 or DNA-PK_cs_ was introduced (Figure 4C,D). These results showed that miR-145-induced reduction of DNA-PKcs expression caused a defect in DNA damage repair and contributed to reduced cell survival after irradiation. Taken together, these results provide evidence that the miR-145-mediated downregulation of DNA-PK_cs_ results in both impairment of DSB repair and increased sensitivity to γ-ray irradiation, which confirmed this study hypothesis regarding the miR-145-DNA-PKcs-NHEJ axis.

### 3.5. miR-145 Overexpression Interferes with Non-Homologous End Joining Repair 

**Figure 5:**
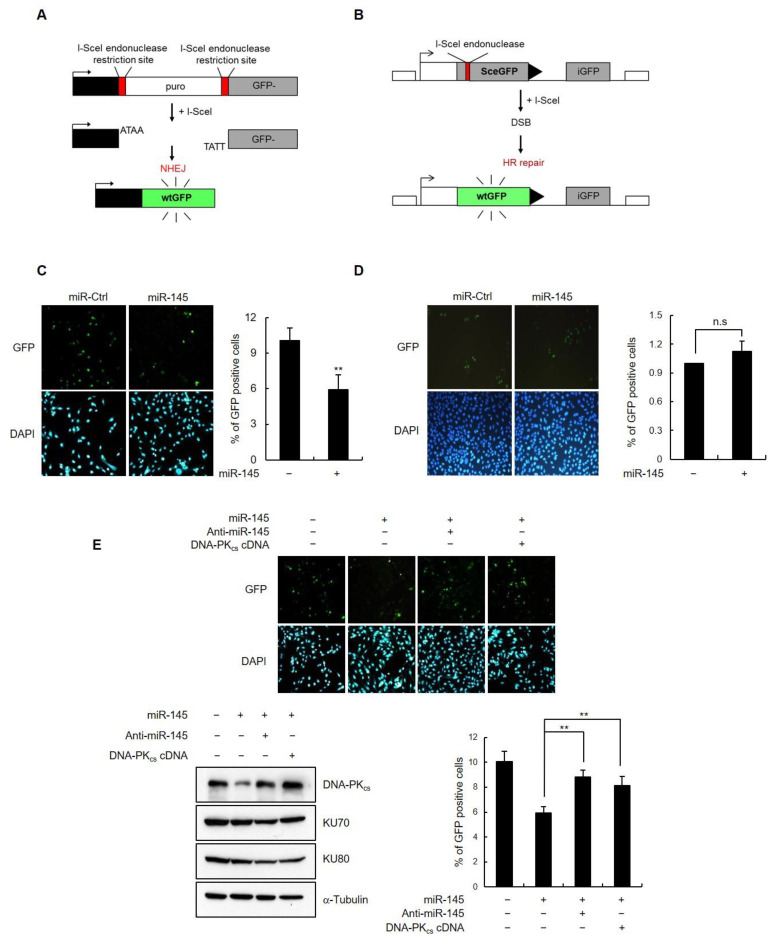
miR-145 affects NHEJ activity. (**A**,**B**) Schematic representation of fluorescence-based NHEJ (**A**) and HR (**B**) assay system using HeLa-EJ5 cells and U2OS-DR-GFP cells, respectively. (**C**,**D**) Cells were transfected with either miR-145 or a control miRNA, followed by transfection with the I-SceI vector. Functional NHEJ (**C**) and HR (**D**) result in GFP-positive cells. The percentage of GFP-positive cells was visualized under a microscope (left) and cells were collected using flow cytometry (right). Results are shown as the mean ± SD (*n* = 3); **, *p* < 0.01. n.s. indicated not statistically significant. (**E**) HeLa-EJ5 cells were co-transfected with miR-145, a control miRNA, anti-miR-145, or DNA-PK_cs_ cDNA in combinations as indicated. After transfection with the I-SceI vector, the cells were analyzed using flow cytometry for efficacy of GFP expression. Results are shown as the mean ± SD (*n* = 3); **, *p* < 0.01.

The two major pathways involved in DNA DSBs repair are homologous recombination (HR) and non-homologous end joining (NHEJ). In the NHEJ pathway, a key player is DNA-PK, which consists of a catalytic subunit (DNA-PK_cs_) and a DNA-binding component (Ku, which is a heterodimer of Ku70 and Ku80). Ku forms a ring-like structure that binds first to the end of the DNA, and then recruits DNA-PK_cs_ and mediates its binding to the DNA [1,2,3,4]. Having observed the effect of miR-145 on both DNA-PK_cs_ expression and DSB repair, we investigated whether miR-145 specifically modulates NHEJ or plays a role in both NHEJ and HR. To address this question, we employed two established assays using HeLa-EJ5 and U2OS-DR-GFP for NHEJ and HR, respectively. In each of these two systems, if the respective HR- or NHEJ-mediated DNA repair occurs, the recombination event generates an intact Green Fluorescent Protein (GFP) allele that is detected and quantified through the level of fluorescence (Figure 5A,B). HeLa-EJ5 and U2OS-DR-GFP cells were transfected with either miR-145 or the control, and then transfected with a vector encoding for I-SceI endonuclease in order to induce DNA damage. The percentage of GFP-positive cells (reflecting the efficiency of either NHEJ-mediated or HR-mediated DNA repair) was assessed 48 h later using flow cytometry. In the presence of miR-145, NHEJ efficiency was markedly decreased, as represented by the low percentage of GFP-expressing cells (Figure 5C), and the expression of the DNA-PK_cs_ protein was accordingly downregulated (Figure S5A). In contrast, miR-145 expression had no effect on the number of GFP-positive U2OS-DR-GFP cells (Figure 5D), indicating that miR-145 regulates NHEJ repair but not HR repair. 

To further confirm that miR-145 affects NHEJ, a second assay, which measures the ability of cells to repair a linearized plasmid, was employed [26]. The plasmid used in this assay, pEGFP-Pem-Ad2, contains an EGFP gene that is interrupted by an intron derived from the rat Pem1 gene with an adenoviral exon (Ad2) engineered into the middle. Because this Ad2 exon is flanked on both sides by HindIII restriction endonuclease sites, undigested or partially digested plasmids retain the Ad2 exon, which in turn becomes integrated into EGFP mRNA, resulting in a nonfunctional EGFP protein. Functional EGFP can only be expressed when the Ad2 exon is excised completely by HindIII digestion, followed by the repair of DSBs (Figure S5B). To test the effect of miR-145 on c-NHEJ, the plasmid was linearized by HindIII digestion in vitro and transfected into HeLa or U2OS cells expressing either miR-145 or a control miRNA. As a transfection control, the pDsRed2-N1 plasmid, which expresses the red fluorescent protein DsRed2, was co-transfected. NHEJ activity was assessed by flow cytometry to quantify the percentage of cells expressing both green and red fluorescence. miR-145 overexpression led to a significantly lower green fluorescence in both HeLa (~30%) and U2OS cells (~60%) than in control cells (Figure S6C). Together, these results suggest that the overexpression of miR-145 and the subsequent downregulation of DNA-PK_cs_ suppressed c-NHEJ.

Next, we asked whether the effect of miR-145 on c-NHEJ activity is due to its effect on DNA-PK_cs_. To address this, we used HeLa-EJ5 cells and co-transfected with miR-145 and an anti-miR-145 or miR-145-insensitive DNA-PK_cs_ expression plasmid. The impairment of c-NHEJ activity in the presence of miR-145 alone was significantly reversed when miR-145 expression was decreased or DNA-PK_cs_ was overexpressed, as measured by the percentage of GFP-expressing cells (Figure 5E). This result suggests that a decrease of miR-145 expression or the presence of miR-145-insensitive DNA-PK_cs_ can overcome the observed NHEJ deficiency caused by miR-145. 

### 3.6. Inhibition of Akt1 Downregulates DNA-PK_cs_ Expression via Induction of Endogenous miR-145 Expression

**Figure 6:**
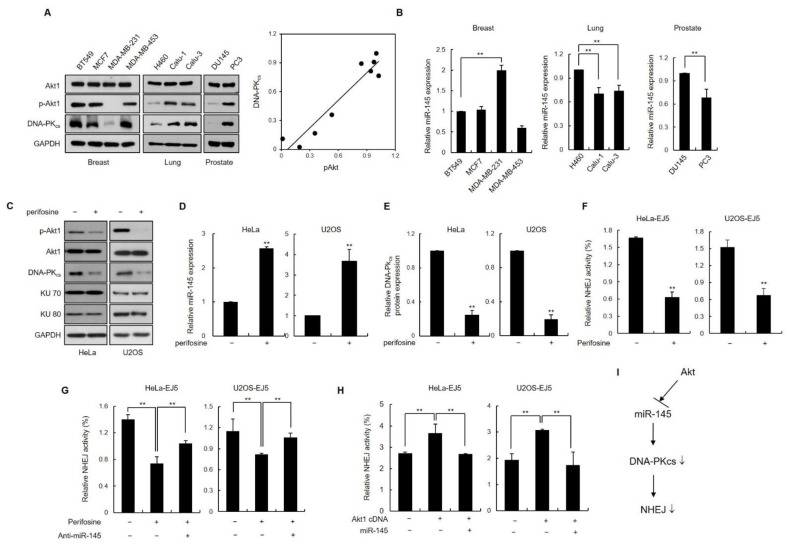
Akt1 inhibition downregulates DNA-PK_cs_ expression through the upregulation of miR-145. (**A**,**B**) Different breast cancer cell lines, BT549, MCF7, MDA MB231, and MDA MB453, lung cancer cell lines, H460, Calu-1, and Calu-3 and prostate cancer cells, DU145 and PC3, were cultured, and the extracts were analyzed for Akt1 activation, DNA-PK_cs_ expression, and miR-145 expression using western blotting (**A**) and qRT-PCR (**B**), respectively. Results are shown as the mean ± SD (*n* = 3); **, *p* < 0.01. A scatter plot showing the correlation between DNA-PKcs expression and Akt activation. (**C**) HeLa and U2OS cells were treated with 50 μM perifosine for 24 h and then cell lysates were subjected to western blotting to measure c-NHEJ factors, DNA-PK_cs_, KU70, and KU80. (**D**) The lysates from the same cohorts of cells described in (**C**) were subjected to RNA extraction, followed by quantitative RT-PCR to measure miR-145 expression. Results were presented as the mean ± SD (*n* = 3); **, *p* < 0.01. (**E**) Quantification of DNA-PK_cs_ western blot signals from three independent experiments as performed in (**C**) using Scion image software. DNA-PK_cs_ protein levels were normalized using Ku70 protein levels (control). Data represent as the mean ± SD (*n* = 3); **, *p* < 0.01. (**F**) EJ5-HeLa and EJ5-U2OS cells were transfected with I-*Sce*I vector and then treated with 50 μM perifosine for 24 h. Yield of GFP-positive cells were determined using flow cytometry. Results are shown as the mean ± SD (*n* = 3); **, *p* < 0.01. (**G**) EJ5-HeLa and EJ5-U2OS cells were co-transfected with anti-miR145 and I-*Sce*I vector and treated with 50 μM perifosine. After 24 h, the percentage of cells expressing GFP was measured using flow cytometry. Data represent the mean ± SD (*n* = 3); **, *p* < 0.01. (**H**) EJ5-HeLa and EJ5-U2OS cells were co-transfected with Akt1 cDNA (pUSE-myc-Akt1) and miR-145, or Akt1 cDNA alone, and NHEJ activity was measured. Data represent the mean ± SD (*n* = 3); **, *p* < 0.01. (**I**) A schematic representing the regulation of NHEJ by miR-145-DNAPKcs axis. See text for details. NHEJ: non-homologous end joining.

Aberrant activation of the PI3K/Akt pathway is a common defect in various human cancer cells [27]. Previous studies have shown that activated Akt1 stimulates NHEJ repair, which then contributes to chemo- or radio-resistance in some tumor cells [28,29,30,31]. However, the mechanism through which Akt1 facilitates NHEJ DNA repair remains unclear. It has been shown that the inhibition of phosphorylated Akt1 (pAkt1) activates miR-145 expression in human cancer cells [32]. We investigated the potential role of miR-145/DNA-PK_cs_ in Akt1-mediated NHEJ activity. Four breast (BT529, MCF7, MDA-MB-231 and MDA-MB-453), three lung (H460, Calu-1 and Calu-3), and two prostate cancer cells (DU145 and PC3) were investigated for the expression of pAkt1 and DNA-PKcs using western blotting. Breast cancer MDA-MB-231, lung cancer H460, and prostate cancer DU145 cells showed low expression levels of pAkt-1 and DNA-PKcs (Figure 6A). In addition, it was observed that the expression of miR-145 was increased in these cells with low expression of pAkt1, indicating that miR-145 expression is inversely correlated with activated Akt1 in these cancer cell lines (Figure 6B). Importantly, the DNA-PK_cs_ expression levels were significantly lower in the three Akt1-inactivated cell lines than in the other Akt1-activated cell lines (Figure 6A, third panel). A scatter plot showed a significant positive correlation between pAkt1 and DNA-PK_cs_ expression (Figure 6A, right). These results suggest a role for activated Akt1 in the positive regulation of DNA-PK_cs_ expression via miR-145. 

As another way to assess whether Akt1 regulates DNA-PK_cs_ expression, we measured the levels of DNA-PK_cs_ expression in HeLa cells in the presence of the Akt1 inhibitor perifosine, which prevents the recruitment of PI3K/Akt1 to the membrane, a step required for activation [33] (Figure 6C). Perifosine treatment resulted in reduced levels of phosphorylated Akt1 and increased miR-145 expression (Figure 6C,D). Notably, DNA-PK_cs_ expression was lower in cells treated with perifosine (Figure 6C,E). Moreover, when HeLa cells overexpressing Akt1 were transfected with miR-145, we found that increased miR-145 expression was associated with lower levels of DNA-PK_cs_ expression (Supplementary Figure S6A–C). 

The next step was to identify any connections between Akt1 and DNA-PK_cs_ as they function in NHEJ. To this end, we measured the percentage of repaired cells using two assay systems: EJ5 cells and pEGFP-Pem-Ad2. The NHEJ assay revealed that DSB rejoining activity was ~50% lower in the presence of perifosine than in untreated control cells (Figure 6F and Supplementary Figure S6D). The decrease in NHEJ activity after perifosine treatment was recovered by anti-miR145 expression (Figure 6G and Supplementary Figure S6E). Consistent with these results, Akt1-overexpressing cells had increased rates of c-NHEJ repair that returned to control levels when miR-145 was overexpressed (Figure 6H). Taken together, these results suggest that Akt1 plays a role in the upregulation of DNA-PK_cs_ function in c-NHEJ by downregulating miR-145 expression.

## 4. Discussion

In mammalian cells, the most severe form of DNA damage is a DSB, which is a potent trigger of the DNA damage response. This type of DNA damage can arise as a result of exposure to both exogenous agents, such as ionizing radiation and certain genotoxic chemicals, and endogenous stresses, including reactive oxygen species [2]. Regardless of the origin of DSBs, the cell responds by activating different pathways to overcome the insult and repair damage. In mammalian cells, NHEJ is the major process by which DSBs are repaired [34]. In NHEJ, Ku70/80 first senses and binds to the DSB and then quickly recruits DNA-PK_cs_. The interaction between Ku70/80 and DNA-PK_cs_ plays a critical role in NHEJ event [2,34]. It is clear that either depletion of DNA-PK_cs_ or the elimination of DNA-PK_cs_ kinase activity renders cells extremely sensitive to agents that induce DSBs and prevents cells from carrying out normal V(D)J recombination [35,36]. However, detailed regulatory mechanisms underlying the role of DNA-PK_cs_ are not well understood. Here, using a bioinformatic analysis followed by mechanistic studies, we have shown that the miR-145 post-transcriptionally represses DNA-PK_cs_ expression by directly targeting the 3′-UTR of DNA-PK_cs_. Furthermore, we have demonstrated that the ectopic expression of miR-145 leads to effects similar to those that occur when DNA-PK_cs_ is depleted, specifically, accumulation of DSBs, enhanced radiosensitivity, and reduced NHEJ activity.

miRNAs regulate the DNA damage response (DDR) through the transcriptional modulation of DDR-related proteins and are induced by DNA damage [37,38]. miRNAs play a role in regulating the DDR by modulating the expression of protein components of various pathways, such as cell cycle arrest, DNA repair, and apoptosis. In particular, miR-145 has been shown to function as a tumor suppressor in a variety of cancer cell types, including colorectal, gastric, breast, renal, hepatic, and non-small lung cancers [39,40,41,42,43,44,45,46]. In these cancers, the tumor suppressive role of miR-145 is achieved, at least partially, through the regulation of cell cycle-related proteins such as CDK6 [47], CDK2 [48], and cyclin D1 [45], and direct targeting of oncogenes, including N-RAS and VEGF-A [49]. Therefore, miR-145 is an important miRNA involved in cell cycle regulation, proliferation inhibition, and apoptosis induction, suggesting that it participates in the regulation of radiosensitivity in cancer cells. In addition to that, DNA damage can regulate the biogenesis of miRNAs which are involved in transcription, processing, and degradation at the transcriptional level [38]. miRNA expression differs depending on the type of cell and DNA damage stimulus. IR induces the expression of some miRNAs, while others repress or do both these functions [50]. miR145 is also regulated by various types of DNA damage. The expression of miR145 is upregulated in certain cells, downregulated in others, and/or does not change after IR treatment [51]. In contrast, miR145 expression was decreased by cisplatin treatment in ovarian carcinoma cells [52]. We showed that miR-145 expression did not change after treatment with IR, hydroxyurea, or camptothecin, but was decreased by cisplatin treatment as previously reported (Supplementary Figure S7). Here, we provide the first evidence that miR-145 targets DNA-PK_cs_, thereby inhibiting its expression and disrupting NHEJ activity. Our results indicate that miR-145 overexpression lowers levels of DSBs repair in both HeLa and U2OS cells, and that the miR-145-mediated downregulation of DNA-PK_cs_ significantly increases radiosensitivity in these cells. A previous study has demonstrated that another miRNA, miR-101, downregulates the expression of both DNA-PK_cs_ and another protein kinase, ATM, and that upregulation of miR-101 sensitizes the tumor cells to radiation [53]. DNA-PK is a critical regulator of genome integrity and is subjected to multiple layers of regulation. The physiological context in which miR-145 targets DNA-PK remains to be elucidated. 

It is well known that radiotherapy and chemotherapy are widely used therapies for cancer treatment. Unfortunately, some types of cancer are initially insensitive and therefore become resistant to radiotherapy or chemotherapy, weakening the therapeutic efficacy of these treatments. Thus, it has become increasingly important to identify additional drugs or therapeutic methods that enhance their efficacy. Most radiotherapy or chemotherapy methods used for treating cancer rely on DNA damage because most cancer cells have impaired DNA repair and proliferate rapidly compared to normal cells [54,55]. When DNA repair is inhibited, the effectiveness of radiotherapy or chemotherapy improves. Because miRNAs are important endogenous regulators of gene expression in DDR, they have the potential for application as sensitizers in cancer therapy. 

In the present study, we demonstrated that the radiosensitivity of HeLa and U2OS cells was considerably increased by miR-145, as demonstrated by clonogenic cell survival assay after exposure to IR. Furthermore, either inhibition of miR-145 or overexpression of DNA-PK_cs_ completely restored the miR-145-induded radiosensitivity. Recently, it was reported that overexpression of miR-145 sensitizes prostate cancer cells to X-ray radiation [56]. In human papillomavirus (HPV)-positive cervical cancer cells, induction of E6 protein expression significantly reduced p53 and miR-145 expression, leading to reduced chemotherapy-induced apoptosis [57], and overexpression of miR-145 in cervical cancer cells enhanced IR sensitivity [58]. In addition, miR-145 sensitizes colorectal cancer cells to the anticancer drug 5-fluorouracil [59]. Thus, the downregulation of DNA-PK_cs_ mediated by miR-145 may be an important beneficial mechanism in radiotherapy for patients with cancer, and therefore, miR-145 may be a new therapeutic target after radiotherapy in human cancer cells. 

Akt1 is a serine/threonine kinase that is a key downstream target in the signaling pathway mediated by phosphoinositide-3 kinase (PI3K), and plays a pivotal role in the regulation of diverse cellular processes, including cell growth, proliferation, and survival [60]. Aberrant activation of the PI3K/Akt1 pathway is common in a wide range of human cancer cells [27]. Accumulating evidence supports the role of Akt1 as an important modulator of DNA damage checkpoint signaling and DSB repair [61,62,63,64,65]. Aberrant/constitutive Akt1 activation is one of the etiological sources of radioresistance in cancer cells and tumors [28,29,31,66], where enhanced c-NHEJ activity appears to play a key role. However, the precise mechanism by which activated Akt1 influences NHEJ activity remains to be elucidated. It has been reported that activated Akt1 phosphorylates MDM2 and in turn induces p53 degradation [32]. In contrast, the miR-145 promoter has two p53-responsive elements and activated p53 enhances miR145 expression through its effect on the pre-miR-145 promoter [32]. In the present study, we discovered that miR-145 expression levels were significantly lower in some cancer cell lines that have activated Akt1 and that activated Akt1 is positively correlated with DNA-PK_cs_ expression. Because our data demonstrated that modulating miR-145 levels affected c-NHEJ by targeting DNA-PK_cs_, we also looked more closely at NHEJ activity. Our results indicated that constitutively active Akt1 promotes NHEJ activity, whereas an Akt1 inhibitor suppresses NHEJ activity by significantly increasing miR145 expression and decreasing DNA-PKcs expression. Importantly, miR-145 overexpression completely restored the NHEJ activity in cancer cells transfected with the constitutively active Akt1 construct. Thus, the Akt1-mediated increase in DNA-PK_cs_ expression via the downregulation of miR-145 could be an underlying mechanism behind the Akt1-mediated increase in NHEJ activity.

In summary, the current study provides the first evidence of an important link between miR-145-impaired DNA repair and the downregulation of DNA-PK_cs_. Our findings revealed the significance of miR-145 in the regulation of DSBs repair, NHEJ activity, and radiosensitivity. miR-145, which is frequently downregulated in cancer cells with activated Akt1, may play an important role in Akt1-induced radioresistance, at least in part by modulating DNA-PK_cs_ expression. Based on the above results, we suggest the model (Figure 6G); miR145 reduces NHEJ activity by targeting DNA-PKcs, whereas activated Akt1 suppresses the expression of miR145 and increases DNA-PKcs expression, thereby increasing NHEJ activity. Understanding the precise role of miR-145 in DNA repair will not only increase our knowledge of how NHEJ activity is regulated but will further our understanding of miR-145 as a biomarker for predicting IR sensitivity and for use as a potential therapeutic target for radioresistance in cancer therapy. 

## Data Availability

Not Applicable.

## Supplementary Materials

The following are available online at https://www.mdpi.com/article/10.3390/cells11091509/s1, Figure S1: (A) Western blot analysis of DNA-PKcs expression in HEK293T cells 48 h after transfection with the negative control miRNA (miR-Ctrl) or each of the three candidate miRNAs. Ku70 and Ku80 protein levels were measured as negative controls. (B) Expression of DNA-PKcs mRNA in indicated miR-transfected HEK293T cells using real-time qRT-PCR. mRNA levels were normalized using Actin mRNA as an internal control. Data represent as the mean ± SD (*n* = 3); **, *p* < 0.01; Figure S2: Sequence of the entire DNA-PKcs 3′UTR (NM_006904; length: 1205 nt). Predicted miR-145 interaction sites at nucleotides 674 and 696 are underlined; Figure S3: (A,B) HeLa (A) and U2OS (B) cells co-transfected with either miR-145 or miR-Ctrl, as well as either anti-miR-145 or DNA-PKcs cDNA, were irradiated with 10 Gy and fixed for immunofluorescence staining of γ-H2AX at the indicated time points. DAPI was used for nuclear staining. (C) HeLa and U2OS cells transfected with miR-145 or miR-Ctrl were treated with NCS at the indicated doses. After 3 days, MTT assay was performed. Results are shown as mean ± SD (*n* = 3); *, *p* < 0.01; Figure S4: (A) HeLa and U2OS cells were co-transfected with miR-145 and its antagomir in combinations as indicated, and DNA-PKcs protein levels were determined using western blotting (*top*). miR-145 expression was measured in parallel using qRT-PCR (*bottom*). Results are shown as the mean ± SD (*n* = 3); **, *p* < 0.01. (B) HeLa and U2OS cells transfected with either miR-145 or miR-Ctrl were co-transfected with a DNA-PKcs cDNA construct lacking the 3′-UTR and subjected to western blotting to measure the DNA-PKcs protein levels (top). The expression of miR-145 in treated cells was quantified using qRT-PCR (*bottom*). Results are shown as the mean ± SD (*n* = 3); **, *p* < 0.01; Figure S5: (A) HeLa-EJ5 cells were transfected with miR-145, and then transfected with I-*Sce*I vector. After 48 h, the expression levels of DNA-PKcs, Ku70 and Ku80 were determined using western blotting. (B) Schematic depiction of the GFP-based c-NHEJ assay using *Hin*dIII-linearized plasmids; the pEGFP-Pem1-Ad2 plasmid has the EGFP gene interrupted with an intron of the rat *Peml* gene in which an adenoviral exon called Ad2 is inserted. When the two *Hin*dIII sites flanking Ad2 are cut by *Hin*dIII ex vivo, *Ad2* is removed from the plasmid. Subsequent transfection of this linearized plasmid allows for re-sealing of the cut sites by the c-NHEJ repair module and the remaining portion of the *Pem1* intron is spliced out while inside the cells, giving rise to reformation of a functional *EGFP* gene. (C) HeLa and U2OS cells were co-transfected with either miR-145 or miR-Ctrl along with pEGFP-Pem1-Ad2. Yields of GFP-positive cells were assayed using flow cytometry. Results are shown as the mean ± SD (*n* = 3); **, *p* < 0.01; Figure S6: (A,B) HeLa cells were transfected with Akt1 cDNA (pUSE-myc-Akt1 vector) along with either miR-145 or miR-Ctrl. Two days after transfection, the levels of miR-145 (A) and DNA-PKcs mRNA (B) were measured by qRT-PCR. Results are shown as mean ± SD (*n* = 3); *, *p* < 0.01. (C) HeLa cells were cotransfected with Akt1 cDNA and miR-145, and the extracts were analyzed for Akt1 activation, DNA-PKcs, Ku70, and Ku80 expression by western blotting. (D) HeLa cells transfected with pEGFP-Pem1-Ad2 were treated with 50 μM perifosine for 24 h. The yield of GFP-positive cells was determined using flow cytometry. Results are shown as the mean ± SD (*n* = 3); **, *p* < 0.01. (E) pEGFP-Pem1-Ad2-transfected HeLa cells were co-transfected with Akt1 cDNA and miR-145, or with Akt1 cDNA alone, and the percentage of cells expressing GFP was measured using flow cytometry. Data represent the mean ± SD (*n* = 3); **, *p* < 0.01. Asterisk indicates overexpressed myc-Akt1; Figure S7: HeLa cells were treated with 10 Gy IR, 10 mM HU, 1 μM CPT or 10 μM cisplatin. After 3 h, the level of miR-145 was measured using qRT-PCR. Results are shown as the mean ± SD (*n* = 3); **, *p* < 0.01; Table S1: miRNA predicted to bind the 3’UTR of the DNA-PKcs by at least six algorithms; Table S2. Targetscan top14 microRNAs for the DNA-PKcs gene.
