# Supplementary material for: MicroRNA-145 Impairs Classical Non-Homologous End-Joining in Response to Ionizing Radiation-Induced DNA Double-Strand Breaks via Targeting DNA-PKcs
Source: Cells. 2022 Apr 30;11(9):1509. doi: 10.3390/cells11091509 (PMC9102532; doi:10.3390/cells11091509)
Supplement: Supplementary file 1 [file cells-11-01509-s001.zip › cells-1605151-supplementary.pdf]

Supplementary Materials

7 supplementary figures

2 supplementary tables

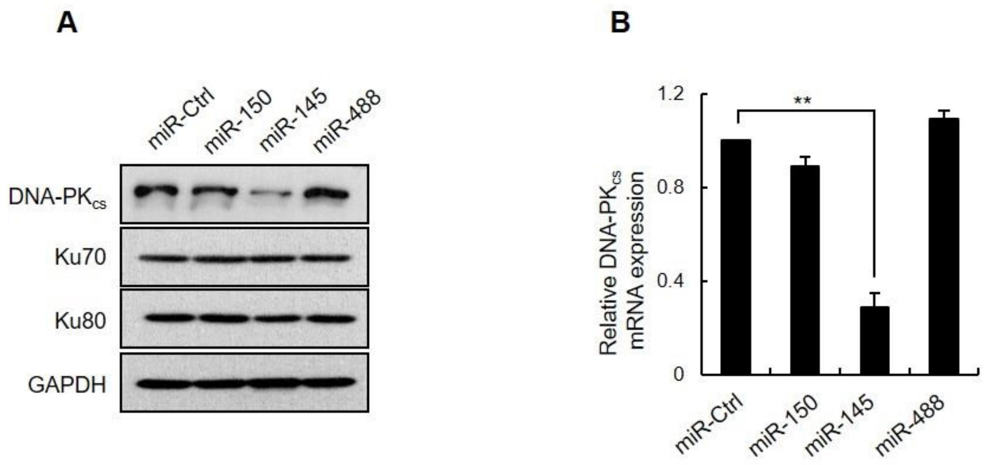

Supplementary Figure S1. (A) Western blot analysis of DNA-PK_{cs} expression in HEK293T cells 48 h after transfection with the negative control miRNA (miR-Ctrl) or each of the three candidate miRNAs. Ku70 and Ku80 protein levels were measured as negative controls. (**B**) Expression of DNA-PK_{cs} mRNA in indicated miR-transfected HEK293T cells using real-time qRT-PCR. mRNA levels were normalized using Actin mRNA as an internal control. Data represent as the mean ± SD (n = 3); **, P < 0.01.

```
   1 GGTCTGTGGG AGTCTGCAGA TAGAAAGCAT TACATTGTTT AAAGAATCTA CTATACTTTG
  61 GTTGGCAGCA TTCCATGAGC TGATTTTCCT GAAACACTAA AGAGAAATGT CTTTTGTGCT
 121 ACAGTTTCGT AGCATGAGTT TAAATCAAGA TTATGATGAG TAAATGTGTA TGGGTTAAAT
 181 CAAAGATAAG GTTATAGTAA CATCAAAGAT TAGGTGAGGT TTATAGAAAG ATAGATATCC
 241 AGGCTTACCA AAGTATTAAG TCAAGAATAT AATATGTGAT CAGCTTTCAA AGCATTTACA
 301 AGTGCTGCAA GTTAGTGAAA CAGCTGTCTC CGTAAATGGA GGAAATGTGG GGAAGCCTTG
 361 GAATGCCCTT CTGGTTCTGG CACATTGGAA AGCACACTCA GAAGGCTTCA TCACCAAGAT
 421 TTTGGGAGAG TAAAGCTAAG TATAGTTGAT GTAACATTGT AGAAGCAGCA TAGGAACAAT
 481 AAGAACAATA GGTAAAGCTA TAATTATGGC TTATATTTAG AAATGACTGC ATTTGATATT
 541 TTAGGATATT TTTCTAGGTT TTTTCCTTTC ATTTTATTCT CTTCTAGTTT TGACATTTTA
 601 TGATAGATTT GCTCTCTAGA AGGAAACGTC TTTATTTAGG AGGGCAAAAA TTTTGGTCAT
 661 AGCATTCACT TTTGCTATTC CAATCTACAA CTGGAAGATA CATAAAAGTG CTTTGCATTG
 721 AATTTGGGAT AACTTCAAAA ATCCCATGGT TGTTGTTAGG GATAGTACTA AGCATTTCAG
 781 TTCCAGGAGA ATAAAAGAAA TTCCTATTTG AAATGAATTC CTCATTTGGA GGAAAAAAAG
 841 CATGCATTCT AGCACAACAA GATGAAATTA TGGAATACAA AAGTGGCTCC TTCCCATGTG
 901 CAGTCCCTGT CCCCCCCCGC CAGTCCTCCA CACCCAAACT GTTTCTGATT GGCTTTTAGC
 961 TTTTTGTTGT TTTTTTTTTT CCTTCTAACA CTTGTATTTG GAGGCTCTTC TGTGATTTTG
1021 AGAAGTATAC TCTTGAGTGT TTAATAAAGT TTTTTTCCAA AAGTAGTGTG TATCTCTTTT
1081 ATGCAGTTTC AAGAGCAAAA TATTCATCTT ACAAAATAGT TTATTATATT ACTTGCCTAG
1141 TTTGTTCTGA TAAATGCGGG TTAAATGCAT TATGGCATTT AAAATAAACA TTTATGGCCA
1201 GGCAC
```

Supplementary Figure S2. Sequence of the entire DNA-PK$_{cs}$ 3'UTR (NM_006904; length: 1205 nt). Predicted miR-145 interaction sites at nucleotides 674 and 696 are underlined.

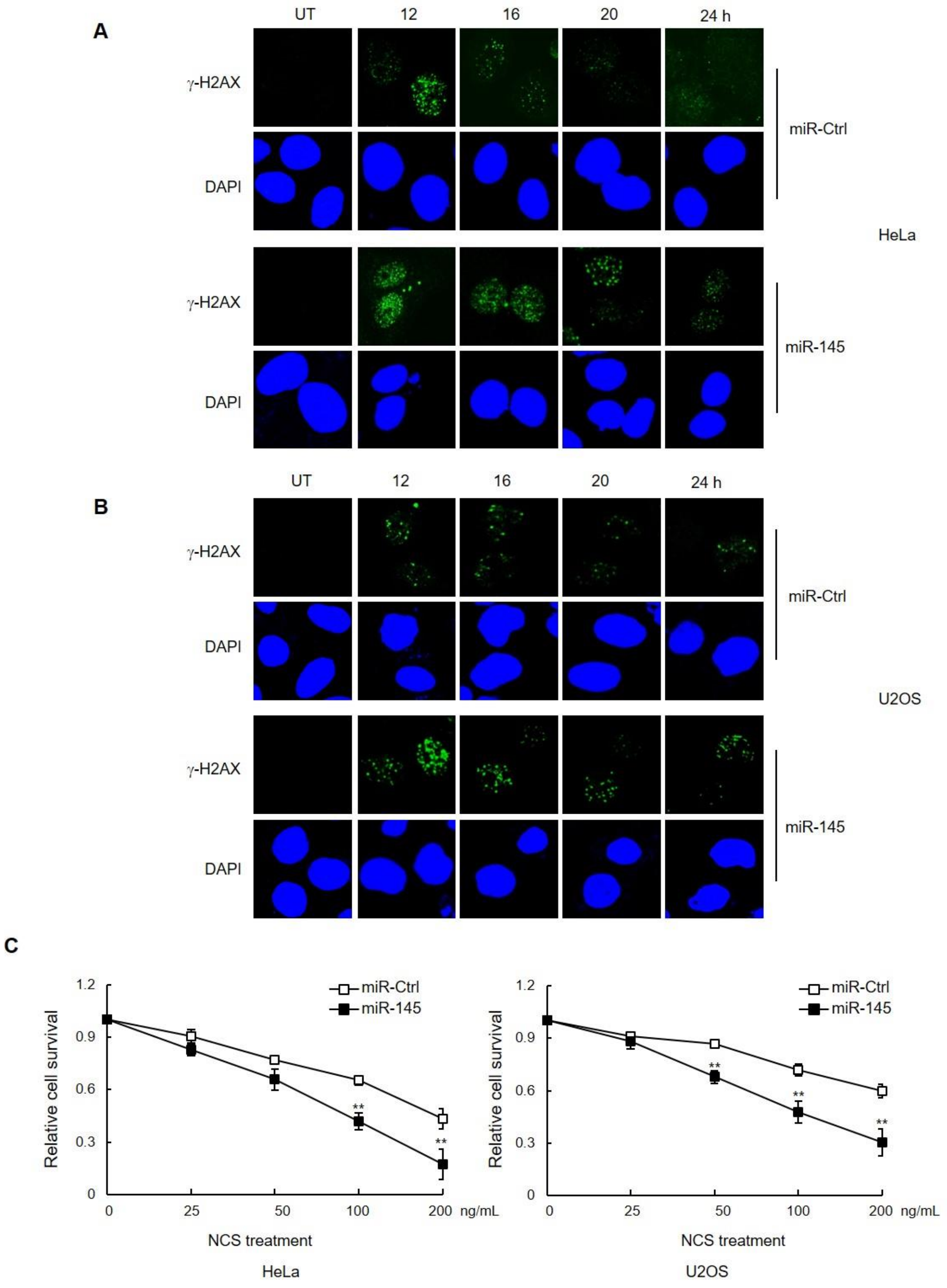

Supplementary Figure S3. (A, B) HeLa (A) and U2OS (B) cells co-transfected with either miR-145 or miR-Ctrl were irradiated with 10 Gy and fixed for immunofluorescence staining of γ-H2AX

at the indicated time points. DAPI was used for nuclear staining. (C) HeLa and U2OS cells transfected with miR-145 or miR-Ctrl were treated with NCS at the indicated doses. After 3 days, MTT assay was performed. Results are shown as mean ± SD (n = 3); **, $P < 0.01$.

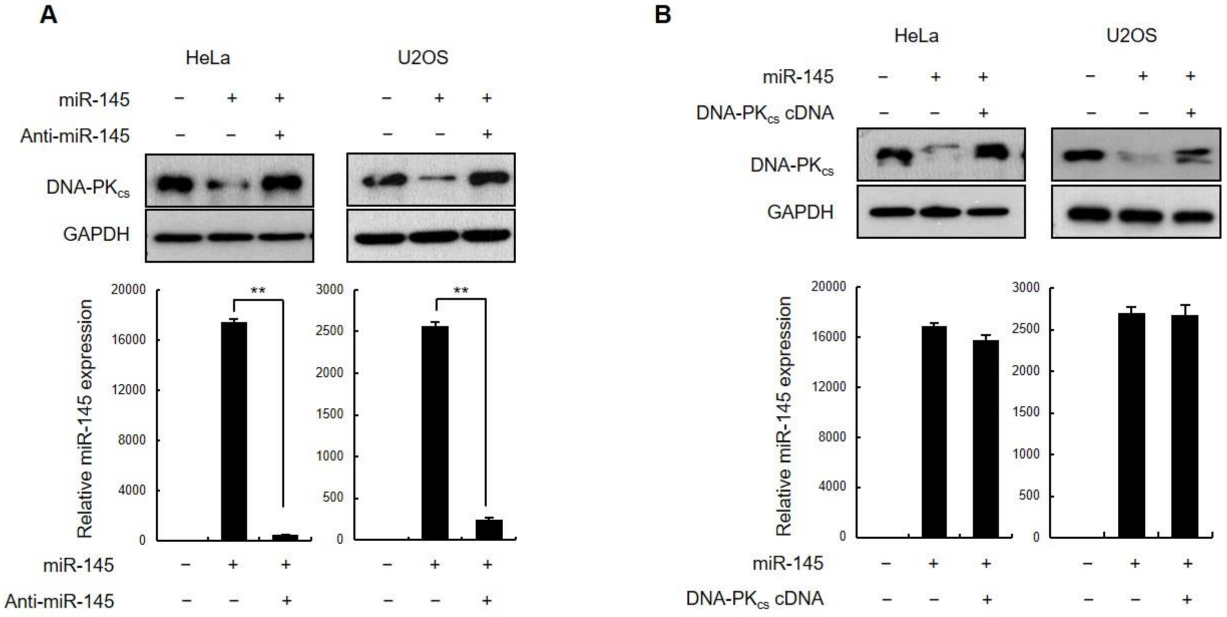

Supplementary Figure S4. (A) HeLa and U2OS cells were co-transfected with miR-145 and its antagomir in combinations as indicated, and DNA-PK_{cs} protein levels were determined using western blotting (*top*). miR-145 expression was measured in parallel using qRT-PCR (*bottom*). Results are shown as the mean ± SD (n = 3); **, $P < 0.01$. (**B**) HeLa and U2OS cells transfected with either miR-145 or miR-Ctrl were co-transfected with a DNA-PK_{cs} cDNA construct lacking the 3′-UTR and subjected to western blotting to measure the DNA-PK_{cs} protein levels (top). The expression of miR-145 in treated cells was quantified using qRT-PCR (*bottom*). Results are shown as the mean ± SD (n = 3); **, $P < 0.01$.

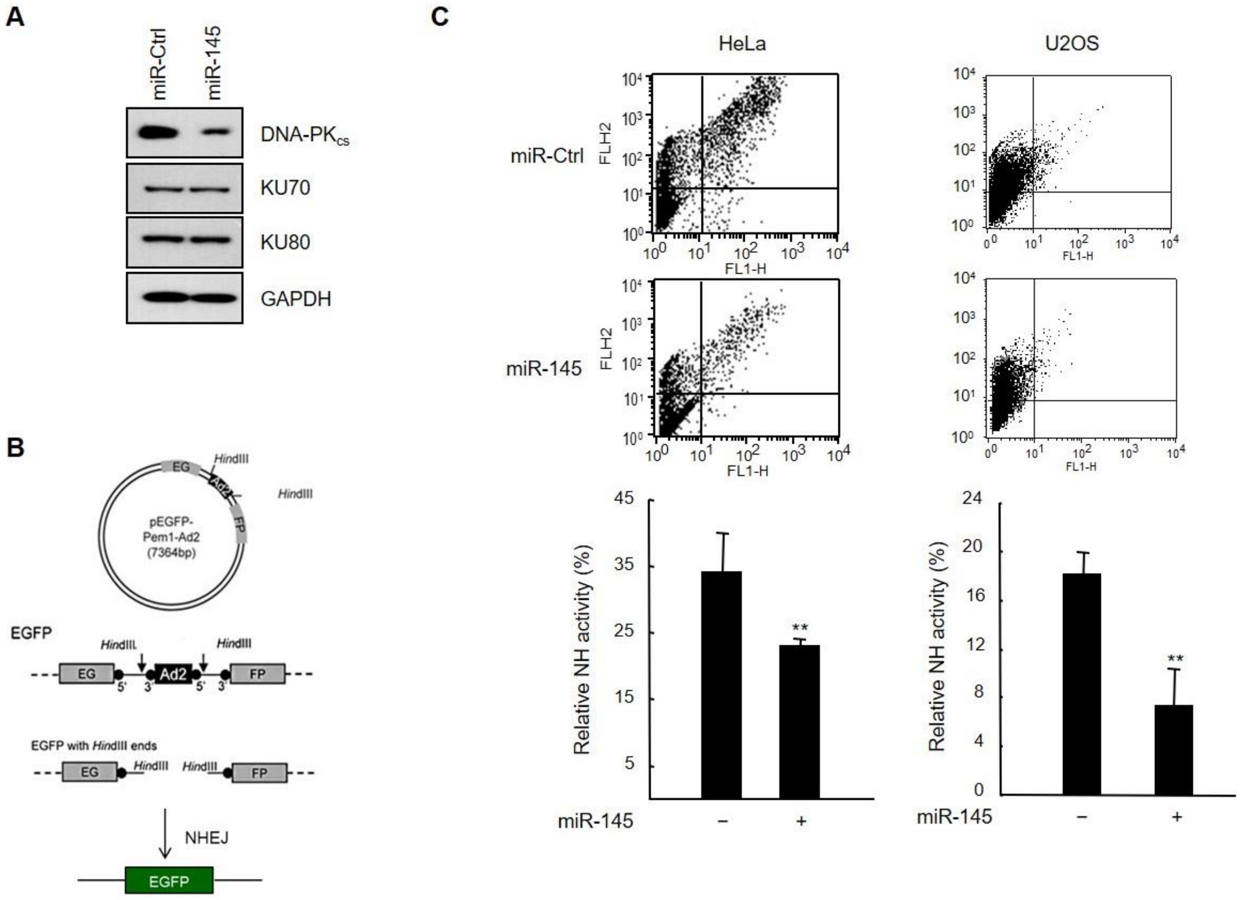

Supplementary Figure S5. (A) HeLa-EJ5 cells were transfected with miR-145, and then transfected with I-*Sce*I vector. After 48 h, the expression levels of DNA-PKcs, Ku70 and Ku80 were determined by Western blotting. **(B)** Schematic depiction of the GFP-based c-NHEJ assay using *Hin*dIII-linearized plasmids; the pEGFP-Pem1-Ad2 plasmid has the EGFP gene interrupted with an intron of the rat *Peml* gene in which an adenoviral exon called Ad2 is inserted. When the two *Hin*dIII sites flanking Ad2 are cut by *Hin*dIII *ex vivo*, *Ad2* is removed from the plasmid. Subsequent transfection of this linearized plasmid allows for re-sealing of the cut sites by the c-NHEJ repair module and the remaining portion of the *Pem1* intron is spliced out while inside the cells, giving rise to reformation of a functional *EGFP* gene. (**C**) HeLa and U2OS cells were co-transfected with either miR-145 or miR-Ctrl along with pEGFP-Pem1-Ad2. Yields of GFP-positive cells were assayed using flow cytometry. Results are shown as the mean ± SD (n = 3); **, *P* < 0.01.

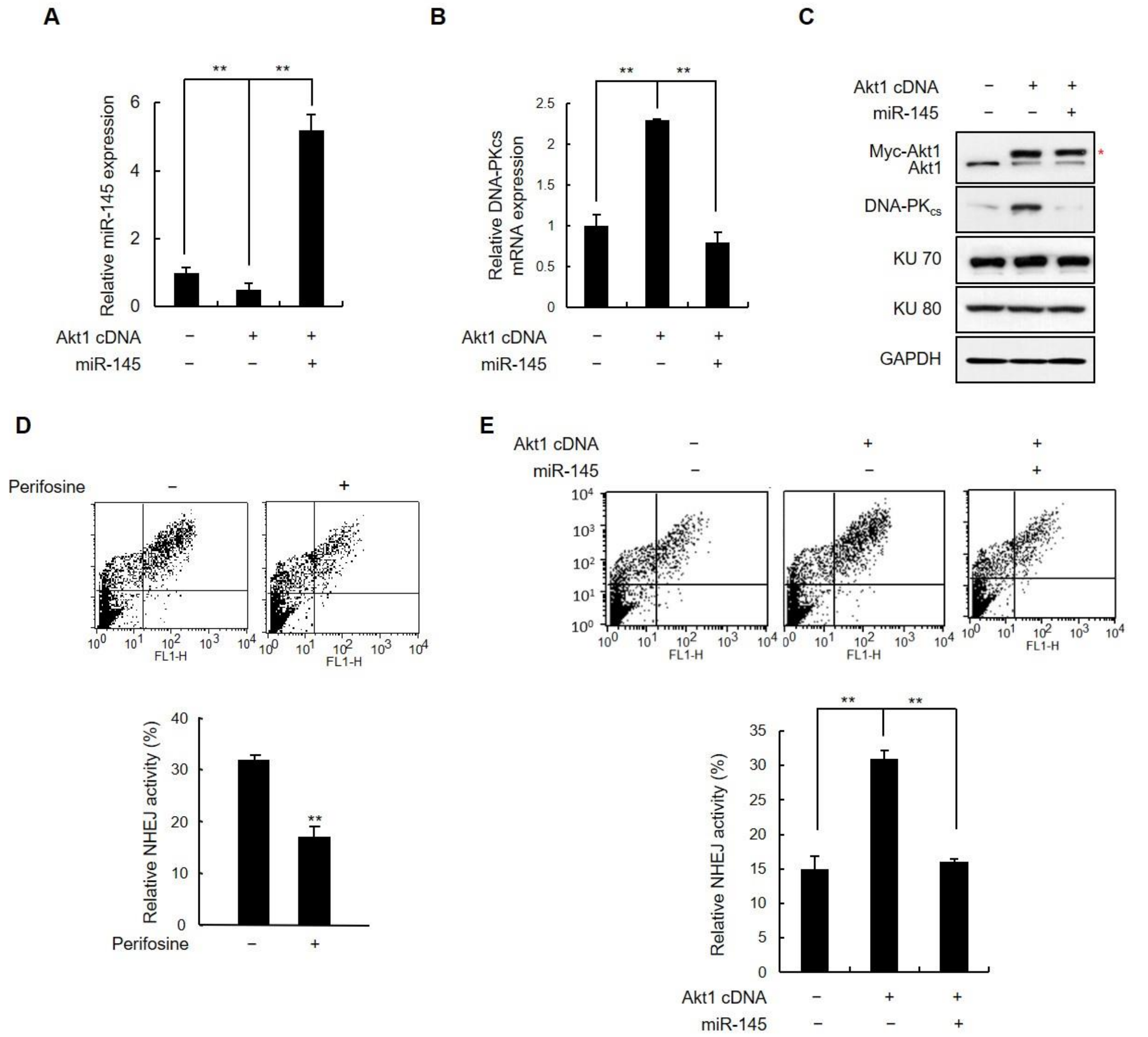

Supplementary Figure S6. (A, B) HeLa cells were transfected with Akt1 cDNA (pUSE-myc-Akt1 vector) along with either miR-145 or miR-Ctrl. Two days after transfection, the level of miR-145 (**A**) and DNA-PK$_{cs}$ mRNA (**B**) were measured using qRT-PCR. Results are shown as mean ± SD (n = 3); **, $P < 0.01$. **(C)** HeLa cells were cotransfected with Akt1 cDNA and miR-145 and the extracts were analyzed for Akt1 activation, DNA-PK$_{cs}$, Ku70 and Ku80 expression using Western blotting. (**D**) HeLa cells transfected with pEGFP-Pem1-Ad2 were treated with 50 μM perifosine for 24 h. The yield of GFP-positive cells was determined using flow cytometry. Results are shown as the mean ± SD (n = 3); **, $P < 0.01$. (**E**) pEGFP-Pem1-Ad2-transfected HeLa cells

were co-transfected with Akt1 cDNA and miR-145, or with Akt1 cDNA alone, and the percentage of cells expressing GFP was measured using flow cytometry. Data represent the mean ± SD (n = 3); **, $P < 0.01$. Asterisk indicates overexpressed myc-Akt1.

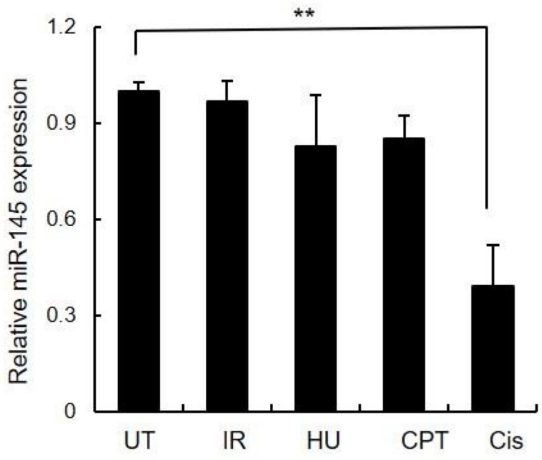

Supplementary Figure S7. HeLa cells were treated with 10 Gy IR, 10 mM HU, 1 μM CPT or 10 μM cisplatin. After 3 h, the level of miR-145 was measured using qRT-PCR. Results are shown as the mean ± SD (n = 3); **, $P < 0.01$.

Supplementary Table S1. miRNA predicted to bind the 3`UTR of the DNA-PK_{cs} by at least six algorithms

microRNA	Algorithms								Number of concordant predictions
	Targetscan	miRWalk	miRanda	miRDB	miRMap	Pictar2	PITA	RNAhybrid	
hsa-miR-145-5p	+	+	+	+	+	-	-	+	6
hsa-miR-150-5p	+	+	+	+	+	-	-	+	6
hsa-miR-488-3p	+	+	+	+	+	-	-	+	6
hsa-miR-4677-5p	+	+	+	+	+	-	-	+	6
hsa-miR-4290	+	+	+	+	+	-	-	+	6
hsa-miR-374c-3p	+	+	+	+	+	-	-	+	6
hsa-miR-1273d	+	+	+	+	+	-	-	+	6
hsa-miR-4699-3p	+	+	+	+	+	-	-	+	6
hsa-miR-4753-3p	+	+	+	+	+	-	-	+	6
hsa-miR-3152-3p	+	+	+	+	+	-	-	+	6
hsa-miR-371b-5p	+	+	+	+	+	-	-	+	6
hsa-miR-3148	+	+	+	+	+	-	-	+	6
hsa-miR-3065-5p	+	+	+	+	+	-	-	+	6
hsa-miR-105-5p	+	+	+	+	+	-	-	+	6
hsa-miR-1252-5p	+	+	+	+	+	-	-	+	6
hsa-miR-3529-3p	+	+	+	+	+	-	-	+	6
hsa-miR-4799-5p	+	+	+	+	+	-	-	+	6
hsa-miR-9-5p	+	+	+	+	+	-	-	+	6
hsa-miR-616-5p	+	+	+	+	+	-	-	+	6
hsa-miR-5009-3p	+	+	+	+	+	-	-	+	6
hsa-miR-373-5p	+	+	+	+	+	-	-	+	6
hsa-miR-3591-5p	+	+	+	+	+	-	-	+	6
hsa-miR-4733-5p	+	+	+	+	+	-	-	+	6
hsa-miR-376a-5p	+	+	+	+	+	-	-	+	6
hsa-miR-3662	+	+	+	+	+	-	-	+	6
hsa-miR-3137	+	+	+	+	+	-	-	+	6
hsa-miR-2116-5p	+	+	+	+	+	-	-	+	6
hsa-miR-5195-3p	+	+	+	+	+	-	-	+	6
hsa-miR-34a-3p	+	+	+	+	+	-	-	+	6
hsa-miR-3123	+	+	+	+	+	-	-	+	6
hsa-miR-3911	+	+	+	-	+	-	-	+	5
hsa-miR-3673	+	+	+	-	+	-	-	+	5
hsa-miR-3646	+	+	+	-	+	-	-	+	5
hsa-miR-548az-5p	+	+	+	-	+	-	-	+	5
hsa-miR-548aj-3p	+	+	+	-	+	-	-	+	5
hsa-miR-548t-5p	+	+	+	-	+	-	-	+	5
hsa-miR-4524a-3p	+	+	+	-	+	-	-	+	5
hsa-miR-518e-5p	+	+	+	-	+	-	-	+	5
hsa-miR-3158-5p	+	+	+	-	+	-	-	+	5

Supplementary Table S2. Targetscan top14 microRNAs for the DNA-PK$_{cs}$ gene

miRNA	conserved sites				poorly conserved sites				Total Context score	Aggregate PCT
	Total	8mer	7mer-m8	7mer-1A	Total	8mer	7mer-m8	7mer-1A		
**miR-488**	0	0	0	0	2	0	1	1	-0.35	< 0.1
**miR-150/5127**	0	0	0	0	1	1	0	0	-0.31	< 0.1
**miR-145**	0	0	0	0	1	1	0	0	-0.29	< 0.1
miR-218/218a	0	0	0	0	2	0	1	1	-0.23	< 0.1
miR-300/381/539-3p	0	0	0	0	1	0	1	0	-0.23	< 0.1
miR-155	0	0	0	0	1	0	1	0	-0.2	< 0.1
miR-9/9ab	0	0	0	0	1	0	1	0	-0.2	< 0.1
miR-455-5p	0	0	0	0	1	0	1	0	-0.17	< 0.1
miR-103a/107/107ab	0	0	0	0	1	0	0	1	-0.17	< 0.1
miR-320abcd/4429	0	0	0	0	1	0	1	0	-0.17	< 0.1
miR-335/335-5p	0	0	0	0	1	0	0	1	-0.15	< 0.1
miR-136	0	0	0	0	1	0	1	0	-0.15	< 0.1
miR-411	0	0	0	0	1	0	0	1	-0.14	< 0.1
miR-873	0	0	0	0	1	0	0	1	-0.13	< 0.1
